# Shifting trends: Detecting changes in cetacean population dynamics in shifting habitat

**DOI:** 10.1371/journal.pone.0251522

**Published:** 2021-05-20

**Authors:** Charlotte Boyd, André E. Punt

**Affiliations:** School of Aquatic and Fishery Sciences, University of Washington, Seattle, WA, United States of America; Swedish University of Agricultural Sciences and Swedish Institute for the Marine Environment, University of Gothenburg, SWEDEN

## Abstract

The ability to monitor population dynamics and detect major changes in population trend is essential for wildlife conservation and management. However, this is often challenging for cetaceans as surveys typically cover only a portion of a population’s range and conventional stock assessment methods cannot then distinguish whether apparent changes in abundance reflect real changes in population size or shifts in distribution. We developed and tested methods for estimating population size and trend and detecting changes in population trend in the context of shifting habitat by integrating additional data into distance-sampling analysis. Previous research has shown that incorporating habitat information can improve population size estimates for highly mobile species with dynamic spatial distributions. Here, using simulated datasets representative of a large whale population, we demonstrate that incorporating individual mark-recapture data can increase the accuracy and precision of trend estimation and the power to distinguish whether apparent changes in abundance reflect changes in population trend or distribution shifts. We recommend that similar simulation studies are conducted for specific cetacean populations to assess the potential for detecting changes in population dynamics given available data. This approach is especially important wherever population change may be confounded with long-term change in distribution patterns associated with regime shifts or climate change.

## Introduction

The ability to monitor population dynamics and detect major changes in population trend is essential for wildlife conservation and management. A reduction in population growth rate or a change in vital rates can provide an early warning of increased extinction risk [e.g., 1–5]. Changes in population growth rates can provide a pertinent metric of the effects of new or increased threats or management actions [e.g., 6, 7]. The timing of changes can point towards specific trigger factors [e.g., 8] or guide research into underlying causes [e.g., 5, 9]. Synchronous changes across taxa may reflect broad changes in environmental conditions or regime shifts [e.g., 9, 10].

Major changes in population trends can be investigated statistically by comparing support for models with or without a change in dynamics [e.g., 2, 8, 11] or evaluating parameters that quantify change [e.g., 6, 12]. Formal statistical approaches are especially useful where a change in trend could have direct policy or management implications (as for species managed under the U.S. Marine Mammal Protection Act, MMPA), and in systems characterized by substantial inherent variation that makes it hard to distinguish long-term trends from random variation. Nevertheless, few studies have attempted this for cetaceans.

Cetacean stock assessments are typically based on distance-sampling data collected during line-transect surveys, but these often have limited statistical power to estimate a single population trend [13], let alone detect a change in trend. The timeframe covered by surveys is typically short relative to the generation length of long-lived species, such as cetaceans. Broad-scale surveys are costly and conducted infrequently. Consequently, surveys often cover only a portion of a population’s range, so that changes in the distribution of habitat can lead to changes in the proportion of the population available for sampling in the survey region [14, 15]. Conventional modeling approaches applied to distance-sampling data [e.g., 16] cannot then distinguish whether an apparent change in abundance in the survey region reflects changes in population size or shifts in distribution. This is especially problematic in the context of oceanographic regime shifts and climate change, which have been associated with distributional shifts for many species [e.g., 17–20].

Previous research has shown that incorporating habitat information can improve estimates of population size for highly mobile species with dynamic spatial distributions [e.g., 15]. Here, we show that additional information may be required to estimate population trends reliably and detect biologically significant changes in population trend in the context of variable distribution patterns.

Mark-recapture analysis of individual photo-identification data provides an alternative to distance-sampling data based on line-transect surveys for assessing population size and survival. Mark-recapture models can be used to detect changes in population dynamics, by incorporating an explicit change in vital rates *a priori* or examining parameter estimates *ex post* [e.g., 4]. Mark-recapture analysis is considered more robust to inter-annual variation in availability for sampling, as it can be inferred that individuals were alive throughout the period from initial mark to final recapture, but is not immune to problems associated with temporary emigration [e.g., 21–23].

Population-level distance-sampling data and individual mark-recapture data are typically analyzed separately, using distinct methodologies. However, results are often difficult to compare, making it hard to reconcile apparent inconsistencies. When comparing estimates of blue whale (*Balaenoptera musculus*) abundance in the California Current Ecosystem, for example, Calambokidis and Barlow [24] questioned whether estimates based on distance-sampling and mark-recapture data were measuring the same population if some individuals were not available for sampling in the survey region in some years. In a later comparison, Calambokidis and colleagues [25] highlighted the divergence between the apparent sharply declining trend in blue whales in the California Current since the 1990s indicated by distance-sampling analysis and the increasing trend indicated by mark-recapture analysis. They hypothesized that this discrepancy could be the result of blue whales expanding their distribution northwards into areas off British Columbia and in the Gulf of Alaska, leading to a reduction in average density in the main survey region off the U.S. west coast that did not represent an actual population decline [see also 18]. This hypothesis provided the initial motivation for the present study.

Integrated population models (IPMs) that combine data on demographic parameters and population size to estimate the parameters of a single underlying population model can help resolve such inconsistencies. By combining data types, IPMs can also achieve more precise parameter estimates with increased statistical power than separate analyses [26]. Here, we focus primarily on estimates of population trend and the power to detect changes in trend.

Bayesian hierarchical or state-space models (BHMs) provide new opportunities for assessing population size and trend using IPMs [27]. Bayesian IPMs comprise two interlinked sets of equations: state equations describe the relationship between demographic parameters and population size; while observation equations describe the relationship between population size and population counts and between demographic parameters and demographic data [26]. BHMs thus facilitate integrated analysis through the joint maximization of the likelihoods of different types of data, with likelihood providing a common currency [28].

BHMs have recently been used to estimate population dynamics for several cetacean populations using distance-sampling data from line-transect surveys [e.g., 15, 29–32]. The model framework developed by Boyd and colleagues [15] includes linked state equations for the population process and the distribution process as a function of habitat, and forms the basis for the models developed in this study. BHMs have also been used to estimate cetacean population size and, more rarely, trends in population size using individual mark-recapture data from photo-identification studies [e.g., 4, 33, 34]. Some Bayesian IPMs have been developed to estimate cetacean population dynamics using previous estimates of population size and data on incidental mortality in fisheries [28], strandings [3], and individual mark-recapture data [35]. However, to our knowledge, no IPMs have yet been developed to assess cetacean populations or other highly mobile nomadic species based on joint estimation of distance-sampling and individual mark-recapture data.

Our objective was to develop a modeling approach to estimate population trends reliably and detect a change in trend in the context of variation in the distribution of habitat using the types of data typically available for large cetaceans.

We approached this by simulating a set of test datasets designed to represent the population dynamics and distribution patterns of a highly mobile long-lived species with substantial inter-annual variation in the distribution of habitat. The test datasets represent four contrasting scenarios: (A) a stable population combined with random interannual variation in the distribution of habitat; (B) a stable population combined with a shift in the distribution of habitat characteristic of a decadal-scale regime shift or long-term climate change; (C) a stable-then-declining population combined with random variation in habitat; and (D) a stable-then-declining population combined with shifting habitat.

We then simulated collection of distance-sampling and individual mark-recapture data and calf indices from the test datasets through a periodic systematic broad-scale line-transect survey supplemented by more frequent spatially targeted mark-recapture surveys.

We developed a series of Bayesian hierarchical IPMs incorporating the various types of data. Model 1 is a distance-sampling model similar to models developed by Boyd and colleagues [15]; Model 2 is an IPM that integrates distance-sampling data and a calf index compiled from the line-transect survey data; Model 3 is a more sophisticated IPM that integrates distance-sampling data and individual mark-recapture data from the line-transect survey; and Model 4 extends this IPM to incorporate additional mark-recapture data from a separate survey platform, such as dedicated small boat studies.

We applied the series of models to each of the test datasets and then evaluated the results, using standard model comparison techniques and evaluation of change parameters to assess the power of each model to distinguish correctly between the stable and declining population.

In several respects, we chose to keep the simulation simple to facilitate exposition. The need to incorporate additional complexity or measurement error in simulation studies used to support real study design is considered in the Discussion.

## Materials and methods

### Test datasets

We provide an overview of our simulation approach here; please see S1 Appendix for further details.

We simulated a single demographically open but geographically closed population over a 16-year period under two scenarios: a stable population over the study period; and a stable population over the first 8 years, followed by a declining population over the second 8 years.

We simulated the population’s spatial distribution so that it varies interannually as a function of relative habitat suitability based on two scenarios: a scenario with random interannual variation in habitat distribution; and a scenario with a shift in habitat distribution after 8 years. We constructed four contrasting test datasets by combining each of the two population trajectories with each of the two habitat scenarios. In each year, individuals were then distributed among habitat cells as a stochastic function of relative habitat suitability in each cell.

**Fig 1:**
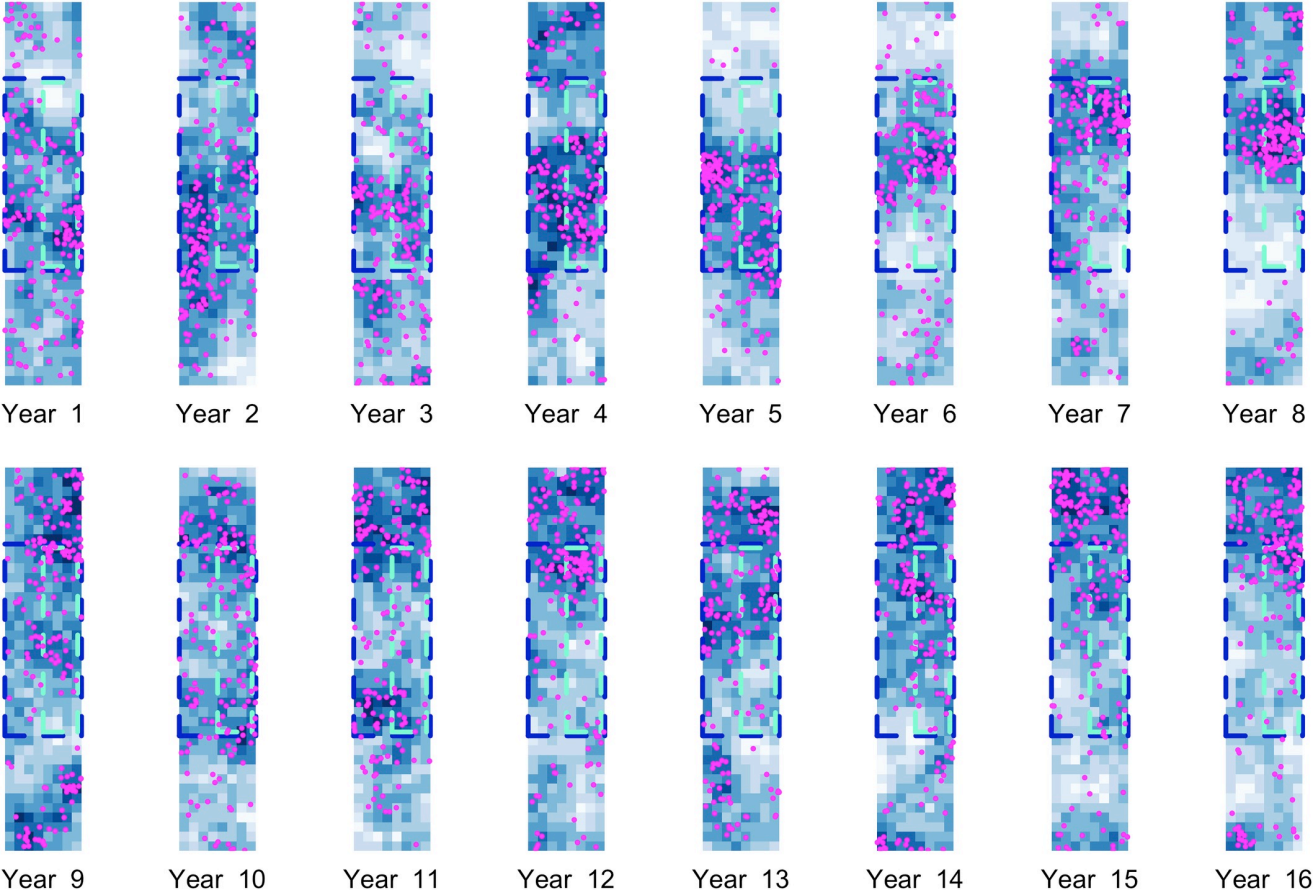
16 representations of a spatially autocorrelated habitat index used to simulate broad-scale population distributions. The sequence shown here is used for the shifting habitat scenarios (Scenarios B and D), with a relatively high proportion of habitat in the survey region in the first 8 years, and a relatively low proportion in the second 8 years. Darker blues indicate more suitable habitat. Magenta points indicate the distribution of a stable population (as in Scenario B) as a function of relative habitat suitability. The survey region is delineated by dark blue dashed lines; the small boat study area (i.e. the area within which target cells are selected) is shown by the cyan dashed lines.

The test datasets were constructed so that the population size and distribution pattern is identical across all four datasets in the first year. Fig 1 shows the evolution of the population distribution pattern over the 16-year study period in Scenario B.

**Fig 2:**
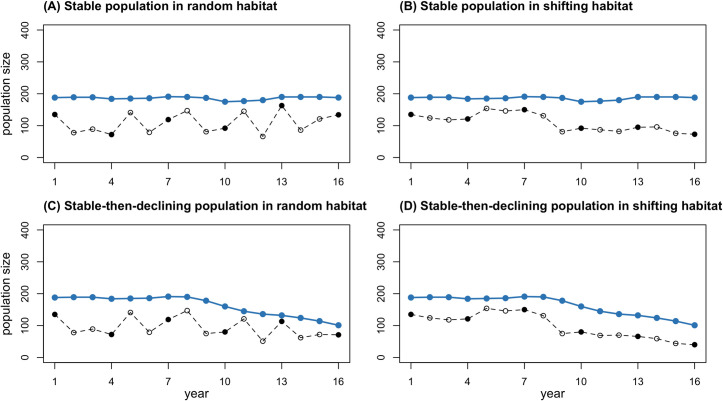
Population trajectories under the four contrasting scenarios over the entire range and in the survey region. Blue points indicate the true population size over the entire range in each scenario. Open black points indicate the number of individuals in the survey region in each scenario, with filled points for survey years.

The survey region encompasses 50% of the total range. The number of individuals in the survey region is relatively stable in Scenario A; exhibits and average decline of approximately 4% per year over the 16-year period in Scenario B, reflecting a shift in habitat distribution; declines by a similar amount in Scenario C, reflecting a true population decline; and declines more steeply in Scenario D, reflecting a combined population decline and habitat shift (Fig 2).

**Fig 3:**
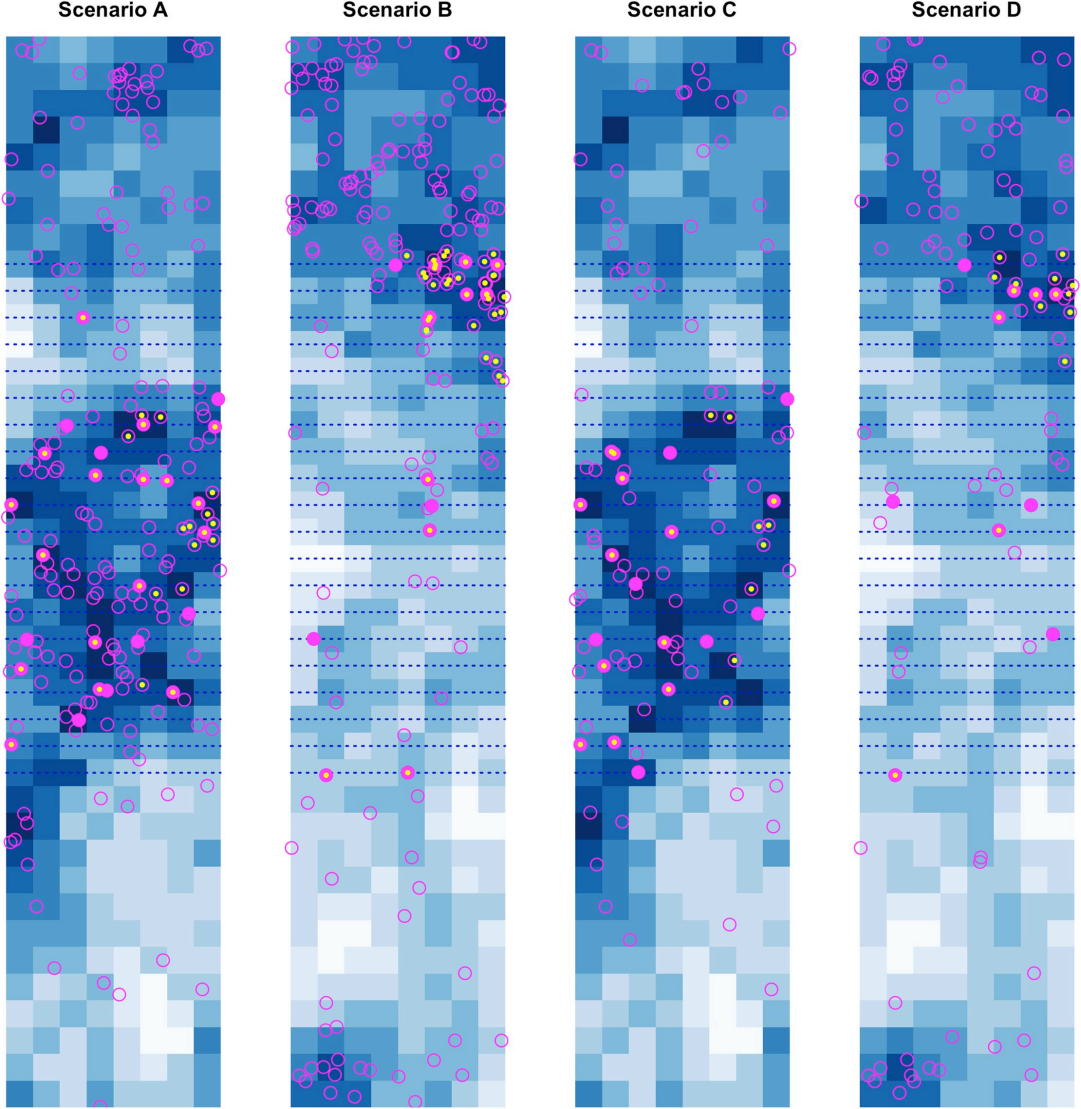
Individual distribution patterns in the final survey year under the four contrasting scenarios. Blue dotted lines indicate transect lines. Filled magenta points indicate individuals detected in the line-transect survey; open magenta points indicate undetected individuals; yellow points indicate individuals identified from either the survey vessel or the small boat.

Fig 3 highlights how the shift in population distribution associated with shifting habitat in Scenarios B and D could undermine efforts to estimate trends based on line-transect data from the survey region. Note that the number of individuals alive is the same in Scenarios A and B and the same in Scenarios C and D.

We simulated collection of distance-sampling data through six systematic line-transect surveys conducted every three years (Fig 3). For simplicity, we set transect lines so that survey effort was the same for each cell in the survey region in each year. The observed data comprise a set of counts of individuals detected along each line-transect segment.

We simulated collection of individual mark-recapture data based on photo-identification from the line-transect survey. In each year, a proportion of adults and juveniles detected during the line-transect survey were identified to individual. All detected whales had the same probability of identification if detected, consistent with identifiability being influenced more by variable sighting conditions than individual distinctiveness. The observed data comprise a set of standard temporal capture histories for individuals identified during line-transect surveys.

We also simulated individual mark-recapture data collected through small boat studies conducted every year in the nearshore section of the survey region (Fig 1). In each year (primary occasion), small boat studies targeted a subset of cells with the most suitable habitat in the nearshore survey region. Each targeted cell was sampled on three secondary occasions in each year based on a robust design framework. The observed data comprise a set of standard temporal capture histories for individuals identified during small boat studies.

We also compiled two calf indices: the number of females with calves among all detected animals in the line-transect survey; and the number of females with calves among all individuals identified in the line-transect survey and small boat studies.

Finally, we extracted a measured habitat covariate for the population’s entire range in each year.

### Integrated population models

The BHMs in this study comprise two linked ecological process submodels: a population process submodel and a distribution process submodel, but differ in how these submodels are configured. They also differ in the types of data used and the associated observation submodels (Table 1).

**Table 1:** Model summaries.

	Model 1a	Model 1b	Model 2	Model 3	Model 4
Population process submodel:					
exponential population model	✓	✓			
binomial demographic model			✓	✓	✓
Distribution process submodel:					
habitat-based		✓	✓	✓	✓
Observation process submodels:					
(a) distances of detected animals	✓	✓	✓	✓	✓
(b) counts of detected animals	✓	✓	✓	✓	✓
(c) calf index data			✓	✓	✓
(d) individual mark-recapture data from line-transect survey				✓	✓
(e) individual mark-recapture data from small boat studies					✓

The observation process in all four models was kept simple to facilitate exposition, consistent with the test datasets. In addition to standard assumptions in distance sampling [36], the models assume perfect detection on the transect line and constant survey conditions.

Two versions of each model were constructed: a baseline version with constant trend and a change-in-trend version with a separate trend parameter (Model 1) or survival parameter (Models 2–4) for the second half of the study period.

#### Model 1: Distance-sampling population model

*Overview*. Model 1 is a distance-sampling population model in Bayesian hierarchical form. It is a simplified version of the integrated population-redistribution model described by Boyd and colleagues [15] and summarized in S2 Appendix.

The dependent data are the perpendicular distances of detected individuals from the transect line, and counts of detected animals (Table 1). The population process submodel is an exponential growth model. In this study, we apply a conventional distance-sampling model without habitat data as a benchmark (Model 1a), and a model with a single dynamic habitat covariate (Model 1b) as described for Model 2 below. The observation process submodel comprises the same likelihoods for distance-sampling data as described for Model 2 below.

#### Model 2: IPM with distance-sampling, calf index, and habitat data

*Overview*. In Model 2, the population process submodel is transformed from an exponential growth model to a binomial demographic model that describes population change as a function of demographic parameters, with the aid of additional data on fecundity derived from the calf index.

The dependent data are same as for Model 1 with the addition of calf index data derived from the line-transect survey (Table 1). The population, distribution, and observation process submodels are described in full below; please see S3 Appendix for notation.

*Population process*. The population process submodel is a simple binomial demographic model based on a single age class comprising all juveniles and adults in the population. (This estimation model is a simplification of the age-structured model that was used to simulate the population.) The population size, *N*_*t*_ in year *t*, is calculated from the population size in the previous year less mortalities, *D*_*t*−1_, plus recruits, *R*_*t*_ (i.e. age-1 juveniles):

$${At\;t=1,\;N}_{t}∼binomial(N_{max},\zeta )$$ (1A)

$${For\;t=2\ldots T:\;D}_{t-1}∼binomial(N_{t-1},1-\varphi )$$ (1B)

$$R_{t}∼binomial(N_{t-1},\rho *\chi )$$ (1C)

$$N_{t}=N_{t-1}-D_{t-1}+R_{t}$$ (1D)

where *ζ* is an inclusion parameter used to sample the initial population size, *N*_*max*_ is the maximum plausible population size, *φ* is average non-calf survival, *ρ* is average fecundity (i.e. calf production per capita), and *χ* is calf survival.

The long-term trend, *υ*, was calculated as a derived parameter in the model to facilitate comparison to the true trend in simulated populations:

υ=log(ρ*χ+φ) (1E)

Two versions of each model were constructed: a baseline version with constant vital rate parameters (as above) and a change-in-trend version with separate estimates of average survival, *φ*, for the first and second halves of the study period:

$$for\;t=2\ldots T_{BP}:\;D_{t-1}∼binomial(N_{t-1},1-\varphi_{1})$$ (1F)

$$for\;t=(T_{BP}+1)\ldots T:\;D_{t-1}∼binomial(N_{t-1},1-\varphi_{2})$$ (1G)

where *T*_*BP*_ indicates the hypothesized change point between periods with different trends.

*Distribution process*. The population is assumed to distribute itself throughout its range in proportion to relative habitat suitability in each survey year following an ideal free distribution [see 15 for further discussion]. The expected proportion of the population in a cell, *p*. *k*_*k*,*t*_, depends on habitat suitability *h* in cell *k* relative to habitat suitability over all *k* = 1…*K* cells in the range:

$${p.h}_{k,t}=exp(b∙{Η}_{k,t})$$ (2A)

$${p.k}_{k,t}={p.h}_{k,t}/\sum_{1}^{K}{p.h}_{k,t}$$ (2B)

where ***H*** is a matrix of habitat covariates and ***b*** is a vector of associated coefficients. Here, we use a single dynamic habitat covariate and estimate a single associated parameter. (Eq 2A differs from the formula used to generate the test datasets (see S1 Appendix). The measured covariate, ***H***, only explains about one third of the variation in simulated distribution patterns; additional variation is attributable to unmeasured habitat covariates or measurement error.)

*Observation process*. Distance-sampling data. The likelihoods for distance-sampling data are based on standard distance-sampling theory [36] as implemented by Boyd and colleagues [15]. Assuming that the distribution of individuals at local scales follows a homogenous Poisson point process and a stationary half-normal detection function, the likelihood of the perpendicular distances, *x*_*j*_, of detected individuals (*j* = 1… *J*) from the transect line is:

$$x_{j}∼normal(0,s^{2})Τ(0,x_{max})$$ (3)

where *T*(0, *x*_*max*_) denotes truncation at 0 and *x*_*max*_. There is one estimated parameter: the variance of the half-normal detection function, *s*^2^.

The likelihood of the number of individuals detected in each surveyed cell in the line transect survey in each year, *n*_*k*,*t*_, is assumed to follow a Poisson distribution:

$$n_{k,t}∼Pois(\lambda_{k,t})$$ (4A)

The expected number of animals detected in each surveyed cell, *λ*_*k*,*t*_, depends on the total population size, *N*_*t*_, derived from the population process submodel, the expected proportion of the population in the cell, *p*.*k*_*k*,*t*_, derived from the distribution process submodel; and the probability of detecting an individual that is present in the cell along the corresponding transect segment, *p*.*d*_*k*,*t*_:

$$\lambda_{k,t}=N_{t}*{p.k}_{k,t}*{p.d}_{k,t}$$ (4B)

The conditional probability of detecting an individual in a cell along a transect segment, *p*.*d*_*k*,*t*_, depends on the segment length, *l*, the effective strip half-width, *ESW*, the cell area, *A*, and the detection probability on the transect line, *g*(0):

$${p.d}_{k,t}=\frac{2∙l∙ESW∙g(0)}{A}$$ (4C)

Here, we assume *g*(0) = 1 for simplicity. The effective strip half-width, *ESW*, is calculated from the probability density of the detected distances, ESW=1f(0) [see 15 for further details].

Calf index data. Model 2 includes a likelihood for the calf index (i.e. the proportion of cow-calf pairs among detected animals) derived from the line-transect survey. The expected proportion of cow-calf pairs among all detected animals is a function of per-capita fecundity, *ρ*, calf survival, *χ*, and non-calf survival, *φ*, given that the number of calves in year *t* is a function of population size in the previous year:

$${n.c}_{(survey)t}=Binomial(\frac{\rho }{\rho *\chi +\varphi },N_{(survey),t})$$ (5)

where *n*.*c*_(*survey*)*t*_ is the number of cow-calf pairs and *N*_(*survey*),*t*_ is the total number of animals detected in the line-transect survey in each year.

#### Model 3: IPM with distance-sampling data, individual mark-recapture data from the line-transect survey, calf index, and habitat data

*Overview*. Model 3 integrates an open population Cormack-Jolly-Seber (CJS) individual mark-recapture model [37–39] for individuals identified in the line-transect survey. The CJS model is based on a state-space framework similar to that developed by Kéry and Schaub [27; see also 40, 41].

The dependent data are the same as for Model 2, with the addition of individual mark-recapture data from the line-transect survey (Table 1). An additional component is incorporated into the population process submodel to account for marked individuals, as described below. Information on individual availability for sampling in the survey region is extracted from the distribution process submodel. A likelihood for the recapture data is incorporated into the observation process submodel.

*Population process*. In the state-space formulation of the CJS model, the state process is summarized in a latent state matrix, *Z*, with dimensions *N*_*marked*_ x *T* and 1s and 0s indicating whether each marked individual, *i*, is alive or not in each year. The CJS model is conditioned on the first capture or mark—only subsequent recapture events are modeled. The *Z*-matrix is therefore completed sequentially for each marked individual from the year when it was first marked and known to be alive, *t*_*marked*,*i*_, as follows:

$$for\;t=t_{marked,i}:\;z_{i,t}=1$$

$$for\;t=(t_{marked,i}+1)\ldots T:\;z_{i,t}∼Bernoulli(\varphi *z_{i,t-1})$$ (6)

A marked individual’s state, *z*_*i*,*t*_, depends on whether it was alive in the previous year, *z*_*i*,*t*−1_, and the average survival rate, *φ*.

*Distribution process*. Individual availability, *α*_(*survey*)*i*,*t*_, is assumed to follow a Bernoulli distribution where the probability of an individual being available for sampling in the survey region, *p*.*α*_(*survey*)*t*_, is the sum of the expected proportion of the population in a cell, *p*.*k*_*k*,*t*_, for all cells in the line-transect survey region (Fig 1):

$$\alpha_{(survey)i,t}∼Bernoulli({p.a}_{(survey)t})$$ (7)

*Observation process*. Mark-recapture data. For each marked individual, *i*, the likelihood of the individual recapture data from the line-transect survey, *y*_(*survey*)*i*,*t*_, is based on the Bernoulli distribution. Conditional on being alive, *z*_*i*,*t*_, and available for sampling in the survey region *α*_(*survey*)*i*,*t*_, the individual recapture probability in the line-transect survey in a given year, *p*.*r*_(*survey*)*t*_, depends on the joint probability of detecting an individual during the line-transect survey, *p*.*d*_(*survey*)*t*_ and then identifying it from the survey vessel, *p*.*id*_(*survey*)*t*_:

$$y_{(survey)i,t}∼Bernoulli({p.r}_{(survey)t}*\alpha_{(survey)i,t}*z_{i,t})$$ (8A)

$${p.r}_{(survey)t}={p.d}_{(survey)t}*{p.id}_{(survey)t}$$ (8B)

The overall probability of detecting an individual in the survey region during the line-transect survey, *p*.*d*_(*survey*)_, is calculated from the total survey effort in the region and the estimated detection function:

$${p.d}_{(survey)t}=\frac{\sum_{k=1}^{K}\left(2∙l∙ESW∙g(0)\right)}{\sum_{k=1}^{K}A}$$ (8C)

The conditional probability of identifying an individual detected during the line-transect survey, *p*.*id*_(*survey*)*t*_, is calculated from the number of individuals identified compared to the number of individuals detected in the line-transect survey in each year and is provided to the model as data.

#### Model 4: IPM with distance-sampling data, individual mark-recapture data from the line-transect survey and small boat studies, calf index, and habitat data

*Overview*. The dependent data incorporated in Model 4 are same as for Model 3, with the addition of individual mark-recapture data from small boat studies (Table 1).

The method for estimating individual availability in the distribution process submodel is reconfigured to estimate availability in the small boat study area as well as the line-transect survey region (see below). An additional likelihood for recapture data from small boat studies is incorporated into the observation process submodel and the likelihood of the calf index data is adjusted to account for all identified individuals.

*Distribution process*. The distribution process submodel is reconfigured to provide information on the availability of individuals for sampling from the small boat. Individuals that are in the line-transect survey region may or may not be in cells targeted by the small boat. We therefore need to estimate the availability of each specific individual *i* for sampling in both the line-transect survey region, *α*_(*survey*)*i*,*t*_, and in cells targeted by the small boat, *α*_(*sb*)*i*,*t*_. This is achieved by sampling from a multinomial distribution as follows:

$$\alpha_{1:3,i,t}∼multinomial(1,{p.a}_{1:3,t})$$ (9)

where *p*.*a*_1,*t*_ is the probability that an individual is in a cell targeted by the small boat (i.e. the sum of *p*.*k*_*k*,*t*_ for all targeted cells in year *t*); *p*.*a*_2,*t*_ is the probability that an individual is in the line-transect survey region but not in a target cell (i.e. the sum of *p*.*k*_*k*,*t*_ for all cells in the survey region excluding cells targeted by small boat studies in year *t*); and *p*.*a*_3,*t*_ is the probability that an individual is not in the line-transect survey region (i.e. the sum of *p*.*k*_*k*,*t*_ for all cells outside the survey region). Thus, *α*_(*sb*)*i*,*t*_ = *α*_1,*i*,*t*_ .and *α*_(*survey*)*i*,*t*_ = 1−*α*_3,*i*,*t*_.

*Observation process*. Mark-recapture data. The likelihood of individual recapture data from small boat studies, *y*_(*sb*)*i*,*m*,*t*_, is based on the Bernoulli distribution. Conditional on being alive, *z*_*i*,*t*_, and available for sampling in a cell targeted by the small boat, *α*_(*sb*)*i*,*t*_, the individual recapture probability on each small boat sampling occasion in a given year depends on the probability of detection-and-identification from the small boat, *p*.*r*_(*sb*)_:

$$y_{(sb)i,m,t}∼Bernoulli({p.r}_{\left(sb\right)}*\alpha_{(sb)i,t}*z_{i,t})$$ (10)

where *m* refers to a secondary sampling occasion. The conditional probability of detecting-and-identifying an individual that is in a cell targeted by the small boat on a single occasion, *p*.*r*_(*sb*)_, is assumed constant.

Calf index data. The calf index is based on identified individuals only to avoid double-counting, as the same individual may be identified in small boat studies and detected, but not identified, in the line-transect survey. Eq 5 is modified accordingly:

$${n.c}_{(id)t}=Binomial(\frac{\rho }{\rho *\chi +\varphi },N_{(id),t})$$ (11)

where *n*.*c*_(*id*)*t*_ is the number of identified females with calves and *N*_(*id*),*t*_ is the total number of individuals identified in small boat studies in each year.

### Model implementation and evaluation

#### Model implementation

Models were estimated using Markov chain Monte Carlo (MCMC) sampling, implemented in JAGS [42]. (See S4 Appendix for model code.) For each model, two separate chains were run for 100,000 iterations with a burn-in of 50,000 and thinning rate of 100 to generate a total of 1,000 saved parameter sets.

Prior distributions were generally non-informative, except that bounds on vital rates parameters were set to appropriate values for a long-lived cetacean [see 43]. Specifically, the upper bound for the fecundity rate, *ρ*, was set at 0.2; the lower bound of non-calf survival, *φ*, at 0.8 and calf survival at 0.6; the upper bound of calf survival was delimited by *φ*. (See S5 Appendix for the complete set of prior distributions.)

Convergence was assessed by visual inspection of trace plots and using standard diagnostics, including the Geweke and Gelman-Rubin diagnostic test [44, 45].

#### Evaluation of model ability to estimate population size and trend

When evaluating model ability to estimate population size and trend, we focused primarily on the constant trend version of each model (Table 1) applied to the Scenario B dataset. This dataset, representing a stable population in shifting habitat, presents a greater estimation challenge than Scenario A (i.e. stable population in relatively stable habitat). We did not evaluate other model-scenario combinations in the same way because either the model and scenario were mismatched (e.g., a constant trend model applied to data with a non-constant trend) or parameters were constant for just a short period (e.g., 8 years in Scenarios C and D).

We compared plots of estimated population trajectories to the known truth derived from the simulated dataset to evaluate each model’s ability to reproduce the true population dynamics in this context. We also compared the posterior distributions to the known truth for key parameters (i.e. final population size, population trend, and non-calf survival where estimated) to evaluate the accuracy and precision of parameter estimates.

#### Evaluation of model ability to detect a change in population trend

We used standard Bayesian model comparison techniques and information criteria (specifically, Watanabe’s Information Criterion, WAIC [46, 47]) to evaluate the ability of each model to distinguish correctly between the stable population and stable-then-declining population in all four scenarios. For the purposes of this study, we evaluated a single change point, corresponding to the true transition year. However, in a real study with uncertain transition timing, it would be appropriate to compare models with various plausible change points to identify the transition year with greatest support from the data [see 8 for example].

In addition, we assessed the posterior distribution of the derived change parameter, *Δ* = *υ*_2_−*υ*_1_, in the change-in-trend version of each model to see whether this indicated an estimated negative shift in population dynamics.

## Results

All models converged, as indicated by visual inspection of trace plots and the Geweke and Gelman-Rubin diagnostic tests [44, 45]. The estimated potential scale reduction factors was less than 1.06 for final population size, long-term trend, survival, and deviance in all models.

### Evaluation of model ability to estimate population size and trend

In presenting the results, we focus first on how well the constant trend version of each model performed in estimating population dynamics when challenged by the scenario with stable population and shifting habitat (Scenario B). However, the overall pattern described here, in terms of the relative performance of the various models, was similar across all scenarios.

When evaluating estimates of population size, we focus on the final year as this year is likely to be of greatest interest to managers.

#### Model 1: Distance-sampling model

**Fig 4:**
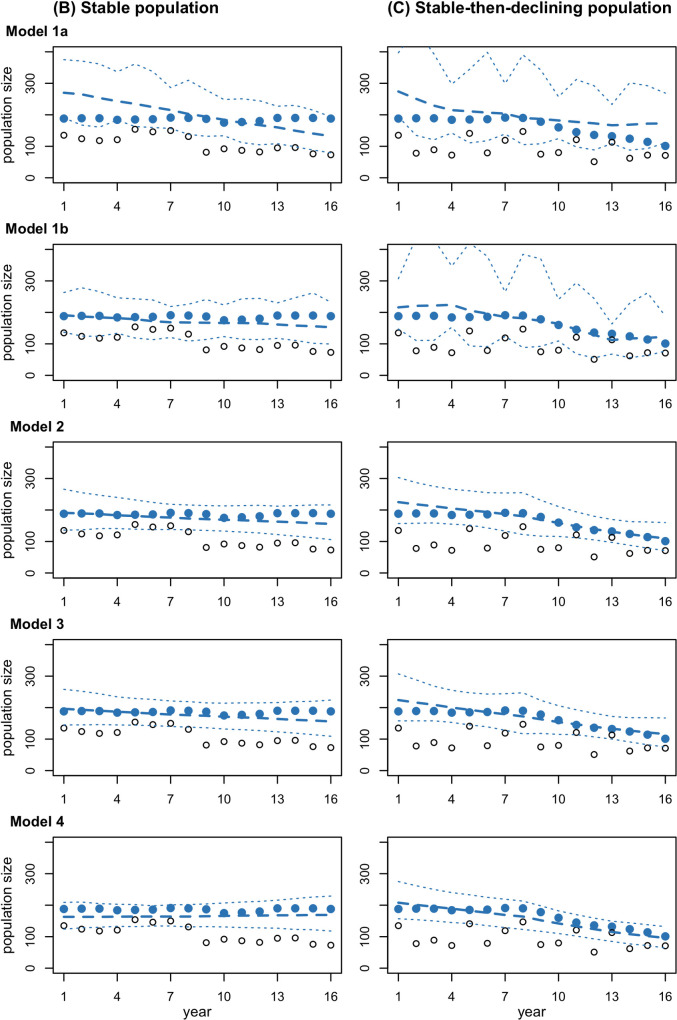
Population processes estimated by Models 1a, 1b, 2, 3, and 4 for Scenarios B (stable population in shifting habitat) and C (stable-then-declining population in randomly varying habitat). Model versions applied to Scenario B assume a constant trend, while those applied to Scenario C allow for a change in trend. Blue points indicate the true population process in each scenario. Open black points indicate the number of individuals in the survey region in each scenario. Blue dashed lines indicate the median of the posterior distribution for population size estimated by each model, and the dotted lines indicate the 95% credible interval.

**Fig 5:**
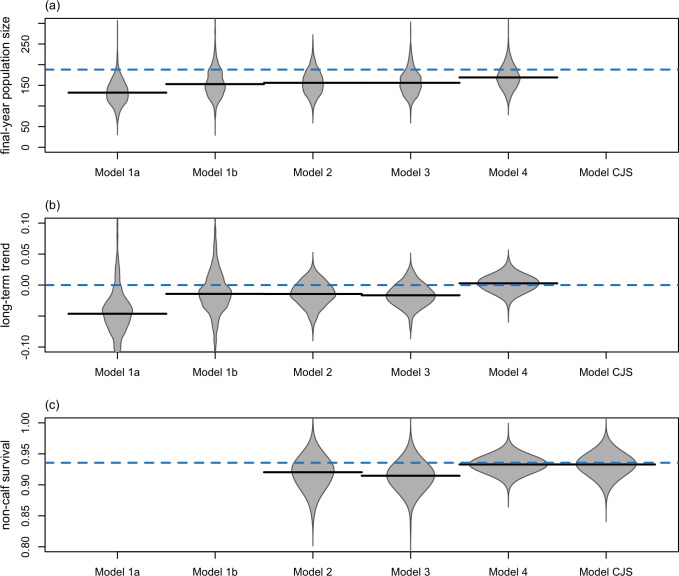
Posterior distributions for (a) final-year population size, (b) long-term trend, and (c) non-calf survival under Scenario B (stable population in shifting habitat). Posterior distributions are shown for Models 1a, 1b, 2, 3, and 4, and a stand-alone CJS model, assuming a stable population. (Non-calf survival is not estimated by Models 1a or b; and final-year population size and long-term trend are not estimated by the CJS model.) In each panel, the blue dashed line indicates the true value, and black horizontal lines indicate the median of the posterior distribution estimated by each model.

The basic distance-sampling population model without habitat data (Model 1a) attributes all interannual variation in numbers of individuals detected in the survey region to variation in population size because it lacks information on variation in the distribution of habitat. When applied to the scenario with shifting habitat, this model attributes the reduction in numbers of individuals observed in the survey region following the habitat shift to a reduction in population size (Fig 4). Consequently, this model estimates a declining trend, even though the population is stable, and underestimates the final population size (Fig 5A and 5B). Broad credible intervals reflect the overall uncertainty about the population process (Fig 4).

In contrast, the distance sampling population model with habitat data (Model 1b) partitions interannual variation in the numbers of individuals observed in the survey region between variation in population size and variation in distribution. This leads to substantial improvements in estimation of the population process (Fig 4), with more accurate estimates of final population size and, especially, long-term trend (Fig 5A and 5B). These results confirm the previous findings of Boyd et al. [15] that incorporating habitat information can improve abundance and trend estimates despite imperfect habitat data.

#### Model 2: IPM with distance-sampling, calf index, and habitat data

The simple IPM in Model 2 is based on a binomial demographic model rather than the exponential population model used in Model 1. The data are the same as for Model 1b, with additional data on fecundity derived from the number of cow-calf pairs sighted during the line-transect survey. The binomial demographic model constrains interannual variation in population size more tightly than the exponential model, leading to more precise estimates of the population dynamics process, especially in non-survey years (Fig 4). Fig 5 shows negligible changes in accuracy but more precise estimates of long-term trend and consequently population size (Fig 5A and 5B). The binomial demographic model also enables estimation of vital rate parameters (e.g., survival, Fig 5C). These results demonstrate that a small amount of additional data on vital rates can be useful if it enables a switch to a binomial demographic model.

#### Model 3: IPM with distance-sampling data, individual mark-recapture data from the line-transect survey, calf index, and habitat data

The IPM with distance sampling and individual mark-recapture data from the line-transect survey (Model 3) provides negligible changes in the accuracy or precision of the estimated population process or parameters (Fig 4). This result reflects the specific parameter values used in this simulation study—in particular, the survey spans a 16-year period, which is short relative to the life span of large whale species, and data are only collected every 3 years with an average capture probability resulting from the combination of availability, detectability and identifiability of 0.08 in each survey year.

#### Model 4: IPM with distance-sampling data, individual mark-recapture data from the line-transect survey and small boat studies, calf index, and habitat data

The IPM with distance sampling and individual mark-recapture data from the line-transect survey and small boat studies (Model 4) led to substantial improvements in the accuracy and precision of parameter estimates. In particular, with additional information on survival, the non-calf survival rate and the long-term trend are both estimated accurately and with greater precision (Fig 5B and 5C). These results demonstrate that incorporating individual mark-recapture data can be extremely useful, even over a relatively short study period for a long-lived species, if data are collected annually with a higher average capture probability (e.g., 0.15 in each year in this study).

Survival estimates were also more precise in the IPM than a stand-alone CJS model applied to the robust design dataset collected in small boat studies (Fig 5C) as distance-sampling analysis provided information on the overall population trend and interannual variation in the availability of individuals for recapture.

### Evaluation of model ability to detect a change in population trend

We now turn to the question of how well the different models perform in terms of detecting a change in population trend.

#### Model comparison

When the constant trend and change-in-trend versions of each model were applied to each of the test datasets, the conventional distance-sampling model without habitat data (Model 1a) fails to diagnose the correct trend in three of four scenarios and indicates only marginally greater support for the correct trend in the fourth scenario (Table 2). The three models with habitat data all performed better than the conventional model. However, only Model 4 (i.e. the IPM with supplementary mark-recapture data from small boat studies) had sufficient information to weight the correct model more highly in all four scenarios. Additional simulations indicated that Model 4’s power to diagnose the correct trend would generally increase with increased mark-recapture sampling effort, as expected (Table 2, final row).

**Table 2:** Model comparison using difference in WAIC; a value of 0 indicates the model with greater support in each model pair; a value of less than 2.00 indicates a model with substantial support.

	Stable population				Stable-then-declining population			
	Random habitat variation (A)		Shifting habitat		Random habitat variation (C)		Shifting habitat	
			(B)				(D)	
Model	Constant trend	Change in trend	Constant trend	Change in trend	Constant trend	Change in trend	Constant trend	Change in trend
1a	0.66	0	0.54	0	0	1.09	0.19	**0**
1b	**0**	2.88	**0**	1.42	0	0.55	2.32	**0**
2	**0**	1.48	0.12	0	0	1.49	0.73	**0**
3	**0**	0.74	**0**	1.85	0	0.28	0	1.18
4	**0**	3.83	**0**	2.71	1.23	**0**	4.22	**0**
4*	**0**	2.12	**0**	4.31	2.32	**0**	5.08	**0**

* Small boat study effort increased by 50%.

Bold values indicate a correct diagnosis.

Focusing on the results for Scenario B (stable population in shifting habitat), the difference in the WAIC was less than 2 for all models except Model 4, indicating similar levels of support from the data. This result was expected as, for all models with habitat data, both model versions should be able to fit data derived from a stable population equally well and there should be limited difference in the number of effective parameters. Model 4 indicated slightly greater support for the constant trend version (i.e. the correct model in this case).

The results were similar for Scenario C (stable-then-declining population in randomly varying habitat). In this case, the difference in the WAIC was less than 2 for all models, and only Model 4 indicated marginally greater support for the change-in-trend version (i.e. the correct model in this case). For all models, both versions had sufficient flexibility to fit the “observed” data well, even though the underlying population exhibited a substantial change in trend.

#### Posterior distribution of the change parameter

**Fig 6:**
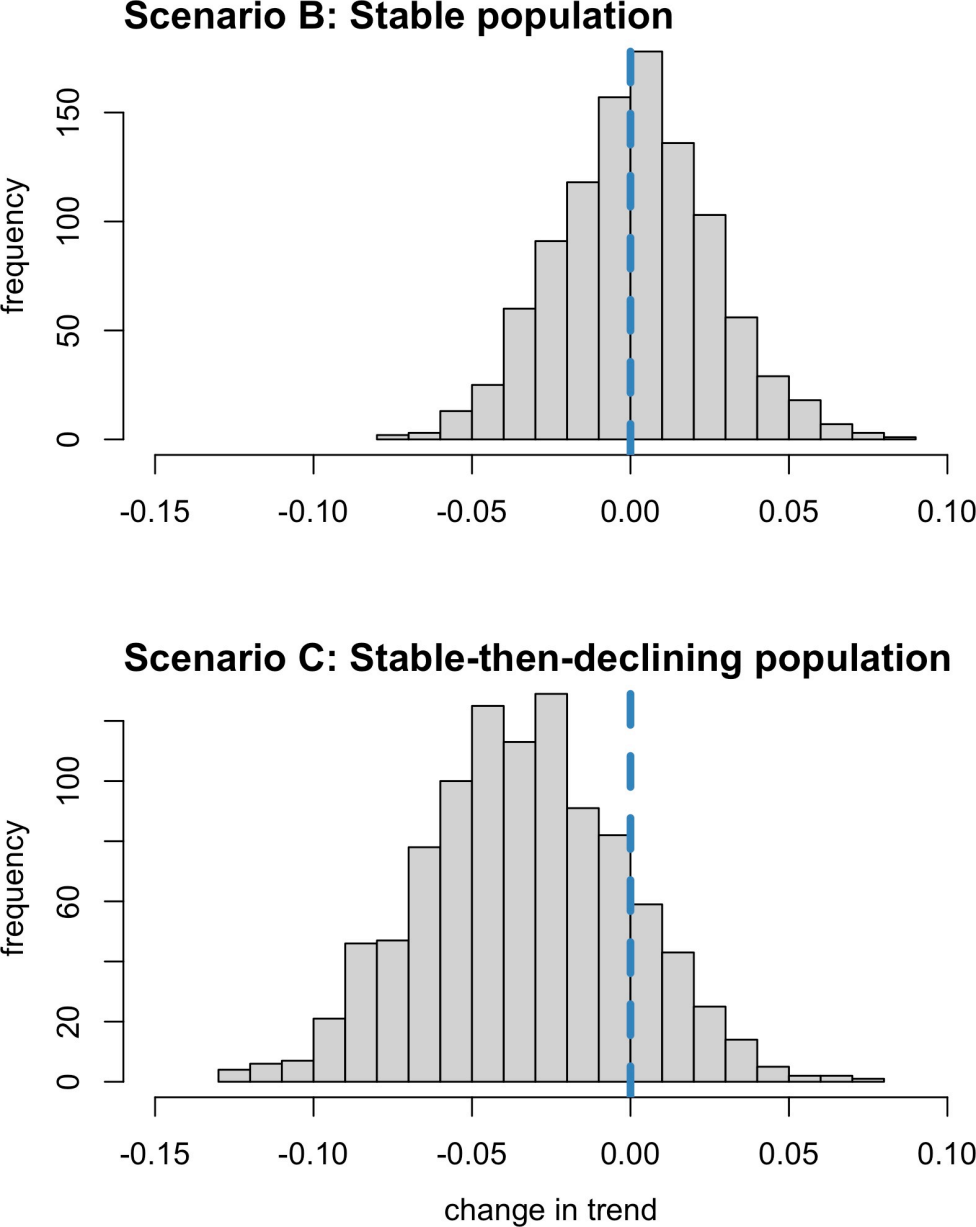
Posterior distributions for change in trend under Scenario B (stable population in shifting habitat) and Scenario C (stable-then-declining population in randomly varying habitat) estimated by Model 4 (change-in-trend version). The blue dashed line indicates no change in trend.

An alternative approach to detecting a change in trend through formal model-based inference is to evaluate the posterior distribution of a parameter that quantifies the hypothesized change. Only Model 4 had sufficient information to estimate the change-in-trend parameter effectively. Fig 6 shows the posterior distribution for this parameter, as estimated by Model 4, based on data derived from a stable population (Scenario B) and a stable-then-declining population (Scenario C). For Scenario B, 46.9% of the posterior distribution was less than 0, indicating approximately 1:1 odds that a positive or negative change occurred in the second half of the study period. In contrast, for Scenario C, 84.9% of the posterior distribution was less than 0, indicating approximately 5:1 odds that a negative change occurred in the second half of the study period.

In this case, the extent of the negative change was underestimated. This likely reflects the short time periods for estimating the old and new trends (8 years), given that Model 4 estimated the consistent trend in Scenario B accurately when given 16 years of data (Fig 6B).

## Discussion

Our objective was to develop and test methods for estimating population trend and detecting changes in trend using simulated data representative of a large whale species. We developed an IPM based on joint analysis of distance-sampling and mark-recapture data that increased the accuracy and precision of trend estimates and power to detect a change in trend compared to separate analyses [see 26 for results from other IPM studies].

Incorporating relevant habitat data provides information on distribution patterns and variation in the proportion of the population that is likely available for detection when surveys cover only a portion of the population’s range. This information enables more accurate population size and trend estimates in the context of variable habitat. Indeed, our results demonstrated that trend estimates can be highly misleading in the case of a long-term habitat shift, if habitat data are not included.

Switching from an exponential population model to a binomial demographic model in an IPM framework provides new information through constraints on interannual population growth that are more biologically realistic for long-lived species with inherently low fecundity, such as cetaceans. This enables more precise estimates of population trend, despite limited additional data (e.g., a calf index derived from just six sampling occasions in this study).

Integrating mark-recapture data into distance-sampling models provides additional information on survival that can lead to more accurate and precise trend estimates depending on sampling effort. In this study, this was achieved through supplementary mark-recapture studies conducted annually from a separate survey platform.

The focus of this study was on trend estimation, but estimation of demographic parameters (e.g., survival and fecundity) can also be important for understanding the demographic causes of population declines and guiding management responses [1, 48]. Integrating distance-sampling data into mark-recapture models provides information on the overall population trend and on interannual variation in availability of individuals for recapture, leading to more precise survival estimates than a stand-alone CJS model.

There are concerns about the potential for over-estimating precision in IPMs where datasets used to estimate different likelihoods are not independent. In this study, for example, an individual detected in the line-transect survey may contribute to both distance-sampling and mark-recapture likelihoods. Ensuring independence of datasets collected in different surveys is considerably more challenging for highly mobile nomadic species than for territorial species [c.f., 49]. We recommend that simulation studies are conducted to evaluate the potential for over-estimation of precision [see 50 for an example] prior to applying the IPMs presented here to real data, especially if the estimated precision could affect management decisions (as in calculation of potential biological removal under the MMPA [51] or application of the International Whaling Commission’s revised management procedure [52] and strike limit algorithms). Note that it would be simple to exclude the mark-recapture data collected from the line-transect survey in Model 4. (These data were included in this study because of concerns about possible sampling bias in mark-recapture data for blue whales off the U.S. west coast as small boat sampling was restricted to the nearshore area [24].)

We were interested in the ability of the various models to detect a change in population trend in the context of shifting habitat given sparse but realistic distance-sampling effort. In this study, only the IPM incorporating supplementary individual mark-recapture data (Model 4) had the power to distinguish between a stable and stable-then-declining population in variable habitat using model comparison techniques given the available data, despite the substantial simulated decline in Scenarios C and D (a one-third reduction in population size over 8 years). These results highlight the challenge of determining whether a change in trend has occurred in a population with variable distribution patterns and limited data. Note that we evaluated a single change point in our analysis of simulated data, but several hypothetical change points might be considered in an estimation study and model comparison used to identify the transition with greatest support.

The question answered by model comparison—*Was there a change in trend*?—may not be useful on its own. It may be more useful to ask—*Was there a biologically significant change in trend*? The posterior distribution of parameters quantifying change can be used to evaluate the weight of evidence for a significant change in trend. In this study, Model 4 indicated posterior odds of approximately 5:1 that a negative change in trend occurred in the stable-then-declining population scenario (C) compared to 1:1 in the stable population scenario (B). It is also possible to estimate the odds that the change in trend exceeded some threshold value. Here, for example, the posterior odds of a negative change in trend greater than 0.02 are approximately 2:1 in the stable-then-declining population scenario (C) compared to 1:4 in the stable population scenario (B).

We focused on large whales in this study, but mark-recapture data is also increasingly available for small cetaceans. We recommend that similar simulation studies are conducted for specific cetacean populations to assess the potential for estimating population size and trend and distinguishing between changes in population dynamics and distributional shifts given available data or to support survey design and decisions about sampling effort. The results would be expected to vary depending on the focal species longevity, the study period, survey effort, detection and recapture probabilities, and other factors.

The models presented here would need several minor modifications for application to real datasets. For example, real line-transect surveys are less regular than depicted here, leading to variation in segment length; diving behavior may lead to imperfect detection on the line; and additional factors such as Beaufort sea-state influence detectability [see 15 for a demonstration of Model 1 applied to real line-transect survey data that addresses these factors]. We anticipate that this approach would provide additional insights into variation in cetacean abundance and the population and distribution processes that underlie any apparent inconsistencies in separate analyses of distance-sampling and mark-recapture datasets, as in the assessments of blue whales off the U.S. west coast that motivated this study [18, 24].

The IPMs presented here could be extended to account for individual heterogeneity in recapture probabilities induced by site-fidelity through spatially-explicit mark-recapture analysis [e.g., 23, 49, 53]. More minor modifications would allow for other sources of individual heterogeneity (e.g., additional random effects could account for individual variation in boat-shyness).

Distance-sampling data derived from line-transect surveys can be used to estimate abundance, trends and distribution patterns over broad spatial scales, but are costly to implement and may only be conducted periodically. Mark-recapture data can often be collected at lower cost and more frequently using smaller vessels, but small boat operations may be spatially restricted, especially in relation to populations that range offshore [e.g., 24]. Our results suggest that IPMs based on a combination of line-transect surveys and supplementary mark-recapture studies may make more efficient use of available data and be more cost-effective than increasing line-transect survey effort. Integrated analysis can combine the broad spatial scales of line-transect surveys with the potential for more frequent but more spatially restricted mark-recapture studies to resolve temporal variation. This approach will be especially useful wherever changes in population dynamics may be confounded with long-term shifts in distribution patterns associated with regime shifts or climate change.

## Supporting information

S1 Appendix Simulation of test datasets. (DOCX)

S2 Appendix Model 1: Distance-sampling population model. (DOCX)

S3 Appendix Notation. (DOCX)

S4 Appendix Model code. (DOCX)

S5 Appendix Prior distributions. (DOCX)
