# Supplementary material for: Shifting trends: Detecting changes in cetacean population dynamics in shifting habitat
Source: PLoS One. 2021 May 20;16(5):e0251522. doi: 10.1371/journal.pone.0251522 (PMC8136736; doi:10.1371/journal.pone.0251522)
Supplement: S1 Appendix — (DOCX) [file pone.0251522.s001.docx]

**S1 Appendix.** Simulation of test datasets

### Population scenarios

We simulated a single demographically open but geographically closed population over a 16-year period under two scenarios: a stable population over the study period; and a stable population over the first 8 years, followed by a declining population over the second 8 years.

Population simulation was based on a stochastic density-independent age-structured Leslie matrix model [1-3], with nine age classes: calves (age 0), juveniles (ages 1-7), and adults (ages 8+). In the first scenario, survival and fecundity parameters were set to simulate a stable population over the study period, with calf survival, $s.c=0.8$, juvenile survival, $s.j=0.9$; adult survival, $s.a=0.95$; and adult female fecundity, $f=0.26$. The second scenario was identical to the first for the first 8 years; juvenile and adult survival were then reduced for the second 8 years (${s.j}_{2}=0.825$; ${s.a}_{2}=0.875$). (See Appendix S2 for notation.)

### Habitat scenarios

We simulated the population’s spatial distribution so that it varies interannually as a function of relative habitat suitability throughout the range. We developed two habitat scenarios: a shifting habitat scenario with a shift in habitat distribution after 8 years; and a random habitat scenario with no temporal autocorrelation in habitat distribution between years.

Both habitat scenarios were developed by simulating numerous representations of a spatially-autocorrelated habitat covariate, $\boldsymbol{H}$, over a fixed range partitioned into equal-area grid cells. We used $\boldsymbol{H}$ plus an additional non-spatially auto-correlated variable, $\boldsymbol{\omega}$, representing unmeasured habitat covariates and/or measurement error, as the basis for simulating the relative habitat suitability, $h_{k,t}$, of each cell (*k* = 1…*K*) in each year (*t* = 1…*T*):

$h_{k,t}=exp(\vartheta_{0}+\vartheta_{1}*H_{k,t}+\omega_{k,t})$ where $\omega_{k,t}\sim normal(0,\Omega)$ (1)

where $\vartheta_{0}$, $\vartheta_{1}$ and $\Omega$ are scalar parameters.

For the shifting habitat scenario, we selected 8 representations in which there was a relatively high proportion of suitable habitat towards the center of the range, followed by 8 representations in which there was a relatively low proportion of suitable habitat towards the center of the range (Fig 1). For the randomly varying habitat scenario, we used the same set of 16 habitat representations, with the same habitat representation in Year 1 and then shuffled the remaining habitat representations at random.

### Individual distribution

We constructed four contrasting test datasets by combining each of the two population trajectories with each of the two habitat scenarios. In each year, individuals (adults and juveniles) were first distributed among cells (i.e. at broad spatial scales) based on a single random draw from a multinomial distribution with probability, $\psi_{k,t}$, defined by relative habitat suitability in each cell:

$\psi_{k,t}={h_{k,t}}/{\sum_{1}^{K} h_{k,t}}$ (2)

A generalized additive model [4] with the number of individuals in each cell as the response variable and a Poisson link function indicated that approximately one third of the deviance in numbers per cell (36.4%) was explained by the measured habitat covariate, $\boldsymbol{H}$. Individuals were then distributed randomly within each cell (i.e. at fine spatial scales) following a homogeneous Poisson point process.

The test datasets were constructed so that the population size and distribution pattern is identical across all four datasets in the first year.

The survey region is located in the center of the range, encompassing 50% of the total range. The number of individuals in the survey region is relatively stable in Scenario A; exhibits an average decline of approximately 4% per year over the 16-year period in Scenario B, reflecting a shift in habitat distribution; declines by a similar amount in Scenario C, reflecting a true population decline; and declines more steeply in Scenario D, reflecting a combined population decline and habitat shift.

### Data collection

The distance-sampling data for blue whales off the U.S. west coast analyzed by Calambokidis and Barlow [5] were derived from line-transect surveys conducted at intervals of 2-3 years or more from Baja California (Mexico) along the U.S. west coast to the Canadian border out to approximately 200 nmi offshore. Survey effort was most intensive off the coast of California. Individual mark-recapture data were also collected along the U.S. west coast each year in dedicated photo-identification studies conducted from small boats operating daily from shore (mostly within 30 nmi). Small boat study locations were targeted to maximize the probability of finding whales and provide broad coverage of coastal areas. Data from the line-transect survey and small boat studies were analyzed separately, except that identification photographs from the line-transect survey were included in the mark-recapture analysis to reduce the nearshore bias of the small boat dataset.

Here, we simulated collection of distance-sampling data through six systematic line-transect surveys conducted every three years (i.e. Years 1, 4, 7, 10, 13, 16). In the shifting habitat scenario, the first three surveys occur during a period of relatively favorable habitat in the survey region, whereas the second three surveys occur during a period of relatively poor habitat in the survey region.

Calves (age 0) were assumed to remain with their mother for their first year of life and were excluded from distance-sampling and mark-recapture data as their detection is not independent from their mother’s. It was assumed that such calves are readily distinguishable from older animals because of their smaller size and that cow-calf pairs are always recognized as such if detected.

***Distance-sampling data from the line-transect survey***

For simplicity, we set transect lines so that survey effort was the same for each cell in the survey region in each year (Fig 3). We calculated the detection probability for each individual (adults and juveniles) as a function of the individual’s distance from the nearest transect line, $x$, based on a stationary half-normal detection function with variance $\sigma^{2},$ censored at a fixed maximum detection distance, $x_{max}$, with certain detection on the line and constant survey conditions. Following standard assumptions in distance sampling [6], each individual could only be detected once, all individuals had the same detection probability given their distance from the nearest transect line, and all distances were measured perfectly. Based on these assumptions, detection of the *j*th individual was based on a random draw from a Bernoulli distribution with probability, $\pi_{j}$, conditional on the individual’s distance from the nearest transect line, $x_{j}$:

$\pi_{j}=exp(\frac{{-x}_{j}^{2}}{{2\sigma}^{2}})$ for $x_{j}\leq x_{max}$ (3)

$\pi_{j}=0$ for $x_{j}>x_{max}$

The observed data comprise a set of counts of individuals detected along each line-transect segment, corresponding to survey effort in each habitat cell. We assumed that the number of individuals detected in each surveyed cell was counted without error, as is standard in distance sampling [6].

***Individual mark-recapture data from the line-transect survey***

We simulated collection of individual mark-recapture data based on photo-identification from the same set of systematic line-transect surveys as the distance-sampling data. In each year, a proportion of adults and juveniles detected during the line-transect survey were identified to individual. All detected whales had the same probability of identification if detected (i.e. there was no individual heterogeneity in identifiability). This is consistent with identifiability being influenced more by variable sighting conditions than individual distinctiveness. We further assumed that the identity of all identified individuals is recorded without error, as is standard in mark-recapture studies [7]. Based on these assumptions, identification of detected individuals in each year was simulated by a random draw from a Bernoulli distribution with probability, $p.id$. The observed data comprise a set of standard temporal capture histories for individuals identified during line-transect surveys (e.g., 0, 1, 0, 0, 1, 0); all-zero capture histories were discarded.

***Individual mark-recapture data from small boat studies***

We also simulated individual mark-recapture data collected from small boat studies conducted every year in the nearshore section of the survey region (Fig 1). In each year (primary occasion), small boat studies targeted a subset of cells with the most suitable habitat in the nearshore survey region, covering approximately 6.25% of the survey region or 3.125% of the range. Each targeted cell was sampled on three secondary occasions in each year based on a robust design framework. Individuals in cells targeted by the small boat were assumed to have stayed within this area for all secondary sampling occasions in each year. This is the standard basis for robust design mark-recapture studies [8], and implies that limited time should elapse between secondary occasions to ensure this is reasonable. For simplicity, we assumed the same detection probability for all individuals in targeted cells and that all detected individuals were identified and their identity recorded without error. Based on these assumptions, detection-and-identification of individuals (adults and juveniles) in cells targeted by the small boat on each secondary occasion was simulated by a random draw from a Bernoulli distribution with probability, ${p.id}_{(sb)}$. The observed data comprise a set of standard temporal capture histories for individuals identified during small boat studies.

***Calf index data***

We compiled two calf indices: the number of females with calves among all detected animals in the line-transect survey, and the number of females with calves among all individuals identified in the line-transect survey and small boat studies.

***Habitat data***

Finally, we extracted the measured habitat covariate, $\boldsymbol{H}$, for the population’s entire range in each year without error.

***References***

1. Leslie PH. On the Use of Matrices in Certain Population Mathematics. Biometrika. 1945;33: 183.
2. Leslie PH. Some Further Notes on the Use of Matrices in Population Mathematics. Biometrika. 1948;35: 213.
3. Caswell H. Matrix population models. Sunderland: Sinauer; 2001.
4. Hastie TJ, Tibshirani RJ. Generalized Additive Models. CRC Press; 1990.
5. Calambokidis J, Barlow J. Abundance of blue and humpback whales in the eastern North Pacific estimated by capture-recapture and line-transect methods. Mar Mamm Sci. 2004;20: 63-85.
6. Buckland ST, Anderson DR, Burnham KP, Laake JL, Borchers DL, Thomas L. Introduction to distance sampling: Estimating abundance of biological populations. Oxford: Oxford University Press; 2001.
7. Kéry M, Schaub M. Bayesian population analysis using WinBUGS: a hierarchical perspective. Waltham: Academic Press; 2012.
8. Pollock KH. A capture-recapture design robust to unequal probability of capture. J Wildl Manage. 1982;46: 752-7.
