# Supplementary material for: Shifting trends: Detecting changes in cetacean population dynamics in shifting habitat
Source: PLoS One. 2021 May 20;16(5):e0251522. doi: 10.1371/journal.pone.0251522 (PMC8136736; doi:10.1371/journal.pone.0251522)
Supplement: S2 Appendix — (DOCX) [file pone.0251522.s002.docx]

**S2 Appendix.** Model 1: Distance-sampling population model

Model 1 is a simplified version of the ideal free extended range model (Model H1b) described by Boyd C, Barlow J, Becker EA, Forney KA, Gerrodette T, Moore JE, Punt AE. Estimation of population size and trends for highly mobile species with dynamic spatial distributions. *Diversity and Distributions*. 2018; 24(1):1-12. (Notation is modified in the summary below to match the main text.)

### Population process submodel

In Model 1, the population process submodel is based on a population-level discrete Markovian exponential growth model:

At *t* = 1, $N_{t}\sim binomial(N_{max}, \zeta)$ (S2.1a)

For *t* = 2…*T*, $N_{t}=N_{t-1}*e^{(\upsilon+\eta_{t})}$ where $\eta_{t}\sim Normal(0, W)$ (S2.1b)

where $N_{t}$ is the number of individuals in year *t*. This submodel has three estimated parameters: initial population size, $N_{1}$; the constant trend, $\upsilon$; and the interannual process variance, $W$. $N_{max}$ is a user-defined upper bound, which should be increased if estimated values approach this limit.

In this study, two versions of Model 1 are fitted: one with constant trend (as in Eq. S2.1b) and the other with a different trend for the first and second halves of the study period:

For *t* = 2…*T_BP_*, $N_{t}=N_{t-1}*e^{(\upsilon_{1}+\eta_{t})}$ (S2.1c)

For *t* = (*T_BP_ + 1)…T*, $N_{t}=N_{t-1}*e^{(\upsilon_{2}+\eta_{t})}$ where $\eta_{t}\sim Normal(0, W)$ (S2.1d)

*T_BP_* indicates the hypothesized change point between periods with different trends.

### Distribution process submodel

In Model 1a, the distribution process submodel is based on the assumption that individuals are distributed randomly throughout the range, such that the proportion of the population expected in any cell is:

${p.k}_{k,t}=1/K$ (S2.2a)

for all years, where $K$ is the total number of cells. There are no estimated parameters in this submodel.

In Model 1b, the distribution process submodel is the same as for Models 2-4, but is also described here for completeness. The population is assumed to distribute itself throughout its range in proportion to relative habitat suitability in each primary sampling period (i.e. survey year) following an ideal free distribution (see Boyd et al., 2018 for further discussion). The expected proportion of the population in a cell, ${p.k}_{k,t}$, depends on habitat suitability $p.h$ in cell *k* relative to habitat suitability over all $k = 1 \ldots K$ cells in the range:

${p.h}_{k,t}=exp(\boldsymbol{b}\cdot\boldsymbol{H}_{k,t})$ (S2.2b)

${p.k}_{k,t}={{p.h}_{k,t}}/{\sum_{1}^{K} {p.h}_{k,t}}$ (S2.2c)

where $\boldsymbol{H}$ is a matrix of habitat covariates and $\boldsymbol{b}$ is a vector of associated coefficients. In this study, we use a single measured environmental covariate and estimate a single associated parameter.

### Observation process submodel

The observation process submodel is based on standard distance-sampling theory (Buckland et al., 2001) as implemented by Boyd et al. (2018). The likelihoods for distances of detected individuals and the number of animals detected in each surveyed cell for Model 1 are the same as for Models 2-4, but are also described here for completeness.

Assuming that the distribution of individuals at local scales follows a homogenous Poisson point process and a stationary half-normal detection function, the likelihood of the perpendicular distances, $x_{j}$, of detected individuals (*j* = 1… *J*) from the transect line is:

$x_{j}\sim normal(0, s^{2})T(0,x_{max})$ (S2.3)

where $T(0,x_{max})$ denotes truncation at 0 and $x_{max}.$ There is one estimated parameter: the variance of the half-normal detection function, $s^{2}$.

The likelihood of the number of individuals detected in each surveyed cell in the line transect survey in each year, $n_{k,t},$ is assumed to follow a Poisson distribution:

$n_{k,t}\sim Pois(\lambda_{k,t})$ (S2.4a)

The expected number of animals detected in each surveyed cell, $\lambda_{k,t}$, depends on the total population size, $N_{t}$, derived from the population process submodel, the expected proportion of the population in the cell, ${p.\kappa}_{k,t}$, derived from the distribution process submodel; and the probability of detecting an individual that is present in the cell along the corresponding transect segment, ${p.d}_{k,t}$:

$\lambda_{k,t}=N_{t}*{p.k}_{k,t}*{p.d}_{k,t}$ (S2.4b)

The conditional probability of detecting an individual that is in a cell along a transect segment, ${p.d}_{k,t}$, depends on the survey effort, i.e. segment length, $l$, the effective strip half-width, $ESW$, the cell area, $A$, and the detection probability on the transect line, *g*(0):

${p.d}_{k,t}=\frac{2\cdot l\cdot ESW\cdot g(0)}{A}$ (S2.4c)

Here, we assume $g(0)=1$ for simplicity. The effective strip half-width, $ESW$, is calculated from the probability density of the detected distances, $ESW=\frac{1}{f(0)}$. This is a simple re-arrangement of the conventional distance-sampling equation for density: $D=\frac{\lambda\cdot f(0)}{2\cdot l\cdot g(0)}$.

Given truncation at $x_{max}$,

$f_{j}\left( 0 \right)=\frac{h\left( 0|0, s^{2} \right)}{\left\{ \Phi(x_{max}|0, s^{2})-0.5 \right\}}$ (S2.4d)

where $h\left( x|0, s^{2} \right)$ is the density at *x* of the normal distribution with zero mean and variance$s^{2}$ (as estimated using Eq. S2.3), and the denominator is the cumulative density of the normal distribution between 0 and $x_{max}$.
