# Supplementary material for: Shifting trends: Detecting changes in cetacean population dynamics in shifting habitat
Source: PLoS One. 2021 May 20;16(5):e0251522. doi: 10.1371/journal.pone.0251522 (PMC8136736; doi:10.1371/journal.pone.0251522)
Supplement: S3 Appendix — (DOCX) [file pone.0251522.s003.docx]

## S3 Appendix. Notation

**Indices (same for test dataset and estimation model)**

*i* = 1…*N_marked_* index for marked individuals

*j* = 1 … *N_max_* index for generic detected individuals

*k* = 1…*K* index for cell

*m* = 1 …*M* index for secondary sampling occasion

*t* = 1…*T* index for year (primary sampling occasion)

*T_BP_* change point year

**Test dataset**

*f* adult female fecundity

$h_{k,t}$ habitat suitability

$H_{k,t}$ habitat covariate

*s.c* calf survival

*s.j* juvenile survival

*s.a* adult survival

*x* perpendicular distance of generic individual *j* from the transect line

$x_{max}$ maximum detection distance

$\vartheta$ habitat suitability parameters

$\pi$ detection probability in line-transect survey

$\sigma^{2}$ variance of the half-normal detection function

$\psi_{k,t}$ probability that an individual occurs in cell *k* in year *t*

$\omega_{k,t}$ unmeasured habitat covariate and/or measurement error

$\Omega$ variance of $\omega_{k,t}$

**Model 1**

$A$ area of a habitat cell

$\boldsymbol{b}$ habitat suitability parameters

$D$ density

$ESW$ effective strip half-width

*f*(0) probability density of detected distances

*g*(0) detection probability on the transect line (assumed to be 1 for this paper)

*h*(*x|*0*, s*^2^) density at *x* of the normal distribution with zero mean and variance *s*^2^

$H_{k,t}$ habitat covariate in cell *k* in year *t*

$l$ length of line transect segment in a habitat cell

$n_{k,t}$ count of animals detected in the line transect survey in cell *k* in year *t*

$N_{t}$ population size

*N_max_* maximum population size

${p.d}_{k,t}$ conditional probability of detecting an animal that is in cell *k* in the line transect survey in year *t*

${p.h}_{k,t}$ habitat suitability of cell *k* in year *t*

${p.k}_{k,t}$ probability that an individual occurs in cell *k* in year *t*

*s^2^* variance of the half-normal detection function

*W* variance of population process

*x_j_* perpendicular distance of generic individual *j* from the transect line

$x_{max}$ maximum detection distance

$\zeta$ inclusion parameter

$\eta_{t}$ population process variation

$\lambda_{k,t}$ expected count of animals detected in the line transect survey in cell *k* in year *t*

$\upsilon$ long-term trend

**Models 2-4**

$\boldsymbol{b}$ habitat suitability parameters

$D_{t}$ deaths

$H_{k,t}$ habitat covariate in cell *k* in year *t*

$n_{k,t}$ count of animals detected in the line transect survey in cell *k* in year *t*

${n.c}_{(id)t}$ number of cow-calf pairs identified in the line transect survey or small boat survey in year *t*

${n.c}_{(survey)t}$ number of cow-calf pairs detected by the line transect survey in year *t*

$N_{t}$ population size

*N_max_* maximum population size

$N_{\left( id \right),t}$ total number of individuals identified in the line transect survey or small boat survey in year *t*

$N_{\left( survey \right),t}$ total number of animals detected in the line transect survey in year *t*

${p.a}_{1:3,t}$ vector of probabilities that an individual is available in the small boat area, elsewhere in the line transect survey region, or not in year *t*

${p.a}_{\left( survey \right)t}$ probability that an individual is available in the line transect survey region in year *t*

${p.d}_{k,t}$ conditional probability of detecting an animal that is in cell *k* in the line transect survey in year *t*

${p.d}_{\left( survey \right)i,t}$ probability of recapturing individual *i* in the line transect survey in year *t,* given that it is in the survey region

${p.id}_{(survey)t}$ observed conditional probability of a detected animal being identified to individual in the line transect survey in year *t*

${p.h}_{k,t}$ habitat suitability of cell *k* in year *t*

${p.k}_{k,t}$ probability that an individual occurs in cell *k* in year *t*

${p.r}_{(sb)}$ conditional probability of detecting and identifying an individual available in the small boat study area on a small boat sampling occasion

${p.r}_{\left( survey \right)i,t}$ conditional probability of detecting and identifying an individual available in the line transect survey in year *t*

$R_{t}$ recruits

*s^2^* variance of the half-normal detection function

*x_j_* perpendicular distance of generic individual *j* from the transect line

$x_{max}$ maximum detection distance

$y_{\left( sb \right)i,m,t}$ indicator of whether individual *i* was detected in the small boat survey on sampling occasion *m* in year *t*

$y_{(survey)i,t}$ indicator of whether individual *i* was detected in the line transect survey in year *t*

$z_{i,t}$ indicator of whether individual *i* is alive in year *t*

$\alpha_{1:3,i,t}$ vector of availability of individual *i* in the small boat area, elsewhere in the line transect survey region, or in neither the small boat area nor the line transect survey region in year *t*

$\alpha_{\left( sb \right)i,t}$ availability of individual *i* to the small boat survey in year *t*

$\alpha_{\left( survey \right)i,t}$ availability of individual *i* to the line transect survey in year *t*

$\zeta$ inclusion parameter

$\lambda_{k,t}$ expected count of animals detected in the line transect survey in cell *k* in year *t*

$\rho$ fecundity rate

$\varphi$ non-calf survival rate

$\chi$ calf survival rate

$\upsilon$ long-term trend
