# Supplementary material for: Shifting trends: Detecting changes in cetacean population dynamics in shifting habitat
Source: PLoS One. 2021 May 20;16(5):e0251522. doi: 10.1371/journal.pone.0251522 (PMC8136736; doi:10.1371/journal.pone.0251522)
Supplement: S4 Appendix — (DOCX) [file pone.0251522.s004.docx]

**S4 Appendix.** Model code

##### Integrated analysis of cetacean population abundance and trends using distance sampling and individual mark-recapture data

#### Charlotte Boyd, Andre E. Punt

## Model 1a: Distance sampling population model without habitat data

model

{

# Priors etc.

zeta ~ dunif(0,1)

u ~ dunif(-0.5, 0.5)

sqrtW ~ dunif(0.00001, 1.5)

tauW <- pow(sqrtW, -2)

s ~ dunif(0.00001,5)

tau <- pow(s, -2)

# Population process submodel

N[1] ~ dbin(zeta, Nmax) # Eq. S2.1a

X[1] <- log(N[1])

for(t in 2:Nyears) {

X[t] ~ dnorm(X[t-1] + u,tauW) # Eq. S2.1b

N[t] <- exp(X[t])

}

# Distribution process submodel

p.k <- 1/Ncells # Eq. S2.2a

# Likelihood of distance data

for(j in 1:Nx) {

x[j] ~ dnorm(0, tau)T(0,x.max) # Eq. S2.3

LL.x[j] <- logdensity.norm(x[j],0,tau)/(pnorm(x.max,0,tau) - 0.5)

}

# Likelihood of count data

ESW <- (pnorm(x.max, 0, tau) - 0.5)/dnorm(0, 0, tau) # Eq. S2.4d

p.d <- (2 * l * ESW)/A # Eq. S2.4c

for(ttt in 1:Nsurvey.years) {

lambda[ttt] <- N[survey.years[ttt]] * p.k * p.d # Eq. S2.4b

for(k in start.survey.cell:end.survey.cell) {

n[k,ttt] ~ dpois(lambda[ttt]) # Eq. S2.4a

LL.n[k,ttt] <- logdensity.pois(n[k,ttt], lambda[ttt])

}} # ttt

}

## Model 1b: Distance sampling population model with habitat data

model

{

# Priors etc.

zeta ~ dunif(0,1)

u ~ dunif(-0.5, 0.5)

sqrtW ~ dunif(0.00001, 1.5)

tauW <- pow(sqrtW, -2)

b ~ dunif(-5,5)

s ~ dunif(0.00001,5)

tau <- pow(s, -2)

# Population process submodel

N[1] ~ dbin(zeta, Nmax) # Eq. S2.1a

X[1] <- log(N[1])

for(t in 2:Nyears) {

X[t] ~ dnorm(X[t-1] + u,tauW) # Eq. S2.1b

N[t] <- exp(X[t])

}

# Distribution process submodel

for(ttt in 1:Nsurvey.years) { for(k in 1:Ncells) {

log(p.h[k,ttt]) <- b * H[k,ttt] # Eq. S2.2b

p.k[k,ttt] <- p.h[k,ttt]/sum(p.h[1:Ncells,ttt]) # Eq. S2.2c

}} # ttt

# Likelihood of distance data

for(j in 1:Nx) {

x[j] ~ dnorm(0, tau)T(0,x.max) # Eq. S2.3

LL.x[j] <- logdensity.norm(x[j],0,tau)/

(pnorm(x.max,0,tau)-0.5)

}

# Likelihood of count data

ESW <- (pnorm(x.max, 0, tau) - 0.5)/dnorm(0, 0, tau) # Eq. S2.4d

p.d <- (2 * l * ESW)/A # Eq. S2.4c

for(ttt in 1:Nsurvey.years) {

pa.survey[ttt] <-

sum(p.k[start.survey.cell:end.survey.cell,ttt])

for(k in start.survey.cell:end.survey.cell) {

lambda[k,ttt] <- N[survey.years[ttt]] *

p.k[k,ttt] * p.d # Eq. S2.4b

n[k,ttt] ~ dpois(lambda[k,ttt]) # Eq. S2.4a

LL.n[k,ttt] <- logdensity.pois(n[k,ttt], lambda[k,ttt])

}} # ttt

}

## Model 2: IPM with distance sampling, calf index, and habitat data

model

{

# Priors etc.

zeta ~ dunif(0,1)

rho ~ dunif(0, 0.2)

phi ~ dunif(0.8, 1)

chi ~ dunif(0.6, phi)

u <- log(rho*chi + phi) # Eq. 1e

b ~ dunif(-5,5)

s ~ dunif(0.00001,5)

tau <- pow(s, -2)

# Population process submodel

N[1] ~ dbin(zeta, Nmax) # Eq. 1a

for(t in 2:Nyears) {

D[t-1] ~ dbin(1-phi, N[t-1]) # Eq. 1b

R[t] ~ dbin(rho*chi, N[t-1]) # Eq. 1c

N[t] <- N[t-1] + R[t] - D[t-1] # Eq. 1d

}

# Distribution process submodel

for(ttt in 1:Nsurvey.years) { for(k in 1:Ncells) {

log(p.h[k,ttt]) <- b * H[k,ttt] # Eq. 2a

p.k[k,ttt] <- p.h[k,ttt]/sum(p.h[1:Ncells,ttt]) # Eq. 2b

}} # ttt

# Likelihood of distance data

for(j in 1:Nx) {

x[j] ~ dnorm(0, tau)T(0,x.max) # Eq. 3

LL.x[j] <- logdensity.norm(x[j],0,tau)/

(pnorm(x.max,0,tau)-0.5)

}

# Likelihood of count data

ESW <- (pnorm(x.max, 0, tau) - 0.5)/dnorm(0, 0, tau) # Eq. S2.4e

p.d <- (2 * l * ESW)/A # Eq. 6

for(ttt in 1:Nsurvey.years) {

for(k in start.survey.cell:end.survey.cell) {

lambda[k,ttt] <- N[survey.years[ttt]] *

p.k[k,ttt] * p.d # Eq. 5

n[k,ttt] ~ dpois(lambda[k,ttt]) # Eq. 4

LL.n[k,ttt] <- logdensity.pois(n[k,ttt], lambda[k,ttt])

}} # ttt

# Likelihood of calf index data

for(ttt in 1:Nsurvey.years) {

nc.survey[ttt] ~ dbin(rho/(rho*chi+phi), N.d[ttt]) # Eq. 7

LL.cv[ttt] <- logdensity.bin(nc.survey[ttt],

rho/(rho*chi+phi), N.d[ttt])

}

}

## Model 3: IPM with distance-sampling data, individual mark-recapture data from the line-transect survey, calf index, and habitat data

model

{

# Priors etc.

zeta ~ dunif(0,1)

rho ~ dunif(0, 0.2)

phi ~ dunif(0.8, 1)

chi ~ dunif(0.6, phi)

u <- log(rho*chi + phi) # Eq. 1e

b ~ dunif(-5,5)

s ~ dunif(0.00001,5)

tau <- pow(s, -2)

# Population process submodel

N[1] ~ dbin(zeta, Nmax) # Eq. 1a

for(t in 2:Nyears) {

D[t-1] ~ dbin(1-phi, N[t-1]) # Eq. 1b

R[t] ~ dbin(rho*chi, N[t-1]) # Eq. 1c

N[t] <- N[t-1] + R[t] - D[t-1] # Eq. 1d

}

for(i in 1:N.id) {

z[i,y.mark[i]] <- 1

for(t in (y.mark[i]+1):Nyears) {

z[i,t] ~ dbern(phi * z[i,t-1]) # Eq. 6

}}

# Distribution process submodel

for(ttt in 1:Nsurvey.years) { for(k in 1:Ncells) {

log(p.h[k,ttt]) <- b * H[k,ttt] # Eq. 2a

p.k[k,ttt] <- p.h[k,ttt]/sum(p.h[1:Ncells,ttt]) # Eq. 2b

}} # ttt

# Likelihood of distance data

for(j in 1:Nx) {

x[j] ~ dnorm(0, tau)T(0,x.max) # Eq. 3

LL.x[j] <- logdensity.norm(x[j],0,tau)/

(pnorm(x.max,0,tau)-0.5)

}

# Likelihood of count data

ESW <- (pnorm(x.max, 0, tau) - 0.5)/dnorm(0, 0, tau) # ~Eq. S2.4e

p.d <- (2 * l * ESW)/A # Eq. 4c

pd.survey <- (2 * l.survey * ESW)/A.survey # Eq. 8c

for(ttt in 1:Nsurvey.years) {

pa.survey[ttt] <- sum(p.k[start.survey.cell:end.survey.cell,ttt])

pr.survey[ttt] <- pd.survey * p.id.survey[ttt] # Eq. 8b

for(k in start.survey.cell:end.survey.cell) {

lambda[k,ttt] <- N[survey.years[ttt]]*p.k[k,ttt]*p.d # Eq. 4b

n[k,ttt] ~ dpois(lambda[k,ttt]) # Eq. 4a

LL.n[k,ttt] <- logdensity.pois(n[k,ttt], lambda[k,ttt])

}} # ttt

# Likelihood of individual recaptures

for(i in 1:N.id) { for(ttt in (y.mark.survey[i]+1):Nsurvey.years) {

a.survey[i,ttt] ~ dbern(pa.survey[ttt]) # Eq. 7

y.survey[i,ttt] ~ dbern(pr.survey[ttt] * a.survey[i,ttt] *

z[i,survey.years[ttt]]) # Eq. 8a

LL.y.survey[i,ttt] <- logdensity.bern(y.survey[i,ttt],

pr.survey[ttt] * a.survey[i,ttt] *

z[i,survey.years[ttt]])

}}

# Likelihood of calf index data

for(ttt in 1:Nsurvey.years) {

nc.survey[ttt] ~ dbin(rho/(rho*chi+phi), N.d[ttt]) # Eq. 5

LL.cv[ttt] <- logdensity.bin(nc.survey[ttt],

rho/(rho*chi+phi), N.d[ttt])

}

}

## Model 4: IPM with distance-sampling data, individual mark-recapture data from the line-transect survey and small boat studies, calf index, and habitat data

model

{

# Priors etc.

zeta ~ dunif(0,1)

rho ~ dunif(0, 0.2)

phi ~ dunif(0.8, 1)

chi ~ dunif(0.6, phi)

u <- log(rho*chi + phi) # Eq. 1e

b ~ dunif(-5,5)

s ~ dunif(0.00001,5)

tau <- pow(s, -2)

pr.sb ~ dunif(0,1)

# Population process submodel

N[1] ~ dbin(zeta, Nmax) # Eq. 1a

for(t in 2:Nyears) {

D[t-1] ~ dbin(1-phi, N[t-1]) # Eq. 1b

R[t] ~ dbin(rho*chi, N[t-1]) # Eq. 1c

N[t] <- N[t-1] + R[t] - D[t-1] # Eq. 1d

}

for(i in 1:N.id) {

z[i,y.mark[i]] <- 1

for(t in (y.mark[i]+1):Nyears) {

z[i,t] ~ dbern(phi * z[i,t-1]) # Eq. 6

}}

# Distribution process submodel

for(t in 1:Nyears) {

for(k in 1:Ncells) {

log(p.h[k,t]) <- b * H[k,t] # Eq. 2a

p.k[k,t] <- p.h[k,t]/sum(p.h[1:Ncells,t]) # Eq. 2b

} # k

pa.survey[t] <- sum(p.k[start.survey.cell:end.survey.cell,t])

pa.sb[t] <- inprod(p.k[1:Ncells,t], sb.cells[1:Ncells,t])

p.a[1,t] <- pa.sb[t]

p.a[2,t] <- pa.survey[t] - pa.sb[t]

p.a[3,t] <- 1 - pa.survey[t]

} # t

# Likelihood of distance data

for(j in 1:Nx) {

x[j] ~ dnorm(0, tau)T(0,x.max) # Eq. 3

LL.x[j] <- logdensity.norm(x[j],0,tau)/

(pnorm(x.max,0,tau)-0.5)

}

# Likelihood of count data

ESW <- (pnorm(x.max, 0, tau) - 0.5)/dnorm(0, 0, tau) # ~Eq. S2.4e

p.d <- (2 * l * ESW)/A # Eq. 4c

pd.survey <- (2 * l.survey * ESW)/A.survey # Eq. 8c

for(ttt in 1:Nsurvey.years) {

pr.survey[ttt] <- pd.survey * p.id.survey[ttt] # Eq. 8b

for(k in start.survey.cell:end.survey.cell) {

lambda[k,ttt] <- N[survey.years[ttt]] *

p.k[k,survey.years[ttt]]*p.d # Eq. 4b

n[k,ttt] ~ dpois(lambda[k,ttt]) # Eq. 4a

LL.n[k,ttt] <- logdensity.pois(n[k,ttt], lambda[k,ttt])

}} # k

for(i in 1:N.id) {

for(t in 1:Nyears) {subregion[i,t]~dcat(p.a[1:3,t])} # Eq. 9

# Likelihood of individual recaptures in line transect survey

for(ttt in (y.mark.survey[i]+1):Nsurvey.years) {

a.survey[i,ttt] <- 1 -

equals(subregion[i,survey.years[ttt]],3)

y.survey[i,survey.years[ttt]] ~ dbern(pr.survey[ttt]*

a.survey[i,ttt]*z[i,survey.years[ttt]]) # Eq. 8a

LL.y.survey[i,ttt]<-

logdensity.bern(y.survey[i,survey.years[ttt]],

pr.survey[ttt] * a.survey[i,ttt] *

z[i,survey.years[ttt]])

} # ttt

# Likelihood of individual recaptures in small boat studies

for(t in (y.mark[i]+1):Nyears) {

a.sb[i,t] <- equals(subregion[i,t], 1)

for(m in 1:N.occasions) {

y.sb[i,m,t] ~ dbern(pr.sb*a.sb[i,t]*z[i,t]) # Eq. 10

LL.y.sb[i,m,t] <- logdensity.bern(y.sb[i,m,t],

pr.sb * a.sb[i,t] * z[i,t])

}} # t

} # i

# Likelihood of calf index data

for(t in 1:Nyears) {

nc.id[t] ~ dbin(rho/(rho*chi+phi), n.id[t]) # Eq. 11

LL.cv[t] <- logdensity.bin(nc.id[t],

rho/(rho*chi+phi), n.id[t])

}

}
