# Supplementary material for: Shifting trends: Detecting changes in cetacean population dynamics in shifting habitat
Source: PLoS One. 2021 May 20;16(5):e0251522. doi: 10.1371/journal.pone.0251522 (PMC8136736; doi:10.1371/journal.pone.0251522)
Supplement: S5 Appendix — (DOCX) [file pone.0251522.s005.docx]

## S5 Appendix. Prior distributions

| Parameter | Prior distribution | Description |
| --- | --- | --- |
| *ξ* | *uniform*(0, 1) | inclusion parameter |
| *υ* | *uniform*(-0.5, 0.5) | long-term population trend |
| $\surd\Omega$ | *uniform*(0.00001, 1.5) | standard deviation of population process variation |
| *ρ* | *uniform*(0, 0.2) | fecundity rate |
| *χ* | *uniform*(0.6, *φ*) | calf survival rate |
| *φ* | *uniform*(0.8, 1) | non-calf survival rate |
| *b* | *uniform(*-5, 5*)* | habitat covariate parameter |
| *s* | *uniform*(0.00001, 5) | standard deviation of the half-normal detection function |
| ${p.r}_{(sb)}$ | *uniform*(0, 1) | conditional probability that an individual available in the small boat study area is detected and identified on a small boat sampling occasion |
